# A Socioecological Framing of the Experiences of Caregivers of Children With Cerebral Palsy in South Africa Post COVID-19

**DOI:** 10.1177/08830738241292844

**Published:** 2024-11-26

**Authors:** Skye Adams, Aneesah Moosa, Razina Bhorat

**Affiliations:** 1 Department of Speech Pathology and Audiology, School of Human and Community Development, University of the Witwatersrand, Johannesburg, South Africa

**Keywords:** cerebral palsy, disability, neurodevelopment, rehabilitation

## Abstract

**Background:**

Post COVID-19, caregivers of children with cerebral palsy in South Africa face unique challenges.

**Methods:**

A qualitative exploratory approach was used. Semistructured interviews were conducted with 14 caregivers of children with cerebral palsy in Gauteng, South Africa. Interviews were audio-recorded, transcribed verbatim, and analysed using thematic analysis.

**Results:**

Lockdown restrictions have had lasting effects on families’ routines and events, reshaping their internal and external functioning. The pandemic introduced new challenges, such as increased physical pain due to the child's weight gain, persistent emotional distress, and a lack of social and governmental support.

**Conclusion:**

Post COVID-19, it is crucial to develop innovative support mechanisms for children with cerebral palsy and their caregivers, focusing on comprehensive health services, robust social support, and targeted interventions to address the ongoing and new challenges faced by these families.

Cerebral palsy is one of the most prevalent causes of childhood disability in South Africa. The condition manifests with neuromuscular spasticity, cognitive dysfunction, behavioral impairments, speech and visual issues, as well as challenges in feeding and gastrointestinal functions.^1^ Consequently, children with cerebral palsy rely on their caregivers, which imposes a significant burden and stress on their caregivers.^2,3^ Research has shown that caregivers of children with cerebral palsy experienced more stress than those of typically developing children during the SARS-CoV-2 (COVID-19) pandemic.^4–6^ In March 2020, like many other parts of the world, South Africa implemented a nationwide lockdown to stop the spread of COVID-19. The sudden onset and unexpected lockdown created and exacerbated existing challenges for children with cerebral palsy because of increased prevalence of underlying health conditions, and the impact on services.^7,8^ As a result of the lockdown, many children were unable to receive health care or attend school, and significant stress was placed on their caregivers who were required to continue with all care at home.^9^ Significant dependence on familial caregiving at home resulted in various detrimental impacts on the caregivers, encompassing aspects of physical, mental, and psychological well-being.^3,10–12^ Research has indicated that the pandemic increased stress, anxiety, and depression among caregivers of children with cerebral palsy.^5,11,13^

In April 2022, the national state of disaster was lifted and all COVID-19 restrictions were removed with the end of the pandemic. However, the difficulties many children and families experienced had long-lasting effects, many that we may not be aware of. Caregivers faced heightened stress, financial strain, and isolation, compounded by job loss and a lack of support.^7,11,13,14^ Because of the long withstanding impact COIVD-19 has had, it is important to explore the ways in which families have transitioned back to their lives and if they require any additional support in their lives and communities.

To fill this gap and understand the transition back to life after the pandemic, the current study used the socioecological model as a conceptual framework to explore caregivers of children with cerebral palsy experiences. Building on the work of Talley and Crews^15^ and that of Bronfenbrenner,^16^ we have applied the socioecological model to assist us in understanding the ways in which caregivers have been affected by the COVID-19 pandemic to inform current research and practice. The theoretical assumption posits that caregiver burden is impacted by the caregiver's characteristics, interpersonal relationships, social interactions, social support, organizational structure, and the environment, as outlined in the socioecological model of health used in this investigation.^17^ According the socioecological model, an individual's health is shaped by multiple layers, encompassing influences at the individual level, interpersonal relationships, social factors, and environmental influences. A multilayered socioecological approach has been used to explore the perceived health and well-being of caregivers^3^ and is being used in the current study to gain insight into the challenges and stressors faced by caregivers of children with cerebral palsy following the COVID-19 pandemic, and to determine factors that may have been overlooked in previous research. Therefore, the purpose of this study was to explore the transition of caregivers of children with cerebral palsy in the South African context post COVID-19 using a socioecological framework.

## Method

This exploratory qualitative study, guided by the principles of fundamental qualitative description, examined caregivers’ experiences and challenges in caring for children with cerebral palsy post COVID-19. Using a qualitative design enabled a rich, clear depiction of these experiences that closely reflects the caregivers’ own words.^18^ This approach is particularly valuable for addressing practical concerns of practitioners and informing policymakers. This study was approved by the Human Research Ethics Committee, Faculty of Humanities of the University of the Witwatersrand (HREC Non-Medical) (approval number: STA_2023_49). All ethical principles were adhered to in the current study, including those related to responsible recruitment, gaining access, and written informed consent.

### Participants

Participants were purposively sampled according to the following inclusion criteria: caregivers had to be 18 years or older and speak English or isiZulu; nevertheless, all participants chose to have their interview conducted in English. The participants interviewed had to be the primary caregiver at home although other family members could be living in the house providing care. All participants had a child (1) diagnosed with cerebral palsy, (2) living at home with the family member during and after the lockdown, (3) aged 4-12 years of age. The wide age range of the child was chosen to approximate school-aged children and to acknowledge the heterogeneity of cerebral palsy and the different organizations that worked with children of different ages. Participants were from the city of Johannesburg and lived in lower- to middle-income socioeconomic neighborhoods.^19^ There was a big disparity regarding income and access in those areas, with the Gini index of Johannesburg being 0.74, highlighting severe inequality (0.5-1).^20^

### Sample Size

As sampling in qualitative research is an iterative process, the sample size was not predetermined. Considering the nature of the topic, and the scope of the study, a sample size of 12-15 participants was estimated to achieve sufficient informational power.

### Data Collection

Data were collected between the dates of May 2023 and August 2023. A list of special education schools and organizations for children with cerebral palsy was collated by the researchers across Gauteng to gain access to the participants. Ten sites were approached, of which only 6 responded. Participant information letters were then sent out by the schools or organizations to all participants who met the inclusion and exclusion criteria and asked to contact the researchers should they wish to participate. Every participant was required to fill out a demographic questionnaire that requested details about the caregiver's age, relationship to the child, employment, and access to public or private health care, and the child's age, gender, and Gross Motor Function Classification System level. An interview guide was developed based on the literature, and the socioecological model, comprising open-ended questions and probes, was used (Supplementary Material S1). The formulation of these questions aligned with the primary research question and associated subquestions, with the intention of delving deeper into the experiences of the caregiver after COVID-19. To ensure the efficacy of the questionnaire and interview guide, a pilot study involving 2 mothers was conducted. Following the completion of the pilot study interviews, no adjustments were made.

Semistructured interviews were conducted with all participants in a single interview. Interviews were conducted by AM and RB, who are speech-language therapists with experience working with children with cerebral palsy. A mutually convenient date and time for an in-person or online interview was scheduled. Participants had a choice of interview location: virtual, their home, or the school. Nine interviews took place online over Zoom, 1 interview took place at the participant's home, and 4 interviews took place at the school. All interviews took place in a quiet room and participants were told that they are able to pause or stop the interview at any given time if necessary. The interviews, averaging about 1 hour each (ranging from 22 to 48 minutes), were audio-recorded and transcribed verbatim by the researchers.

### Data Analysis

Thematic content analysis, following the approach outlined by Braun and Clarke,^18^ was employed to analyze the data. All transcripts were imported into the NVivo computer program for coding and text content analysis. To ensure methodical categorization and coding of the interview transcripts, and maintain the rigor and quality of the analytical process, the 5 steps of qualitative data analysis from Ritchie and Spencer's framework analysis was used as a guide.^21^

The first stage was deductive, line-by-line coding of text using the socioecological framework. For example, codes related to participants’ physical and emotional well-being, emotions, and feelings were coded as individual. The initial coding framework was developed by researchers AM and RB. The authors also included inductive coding to explore new and emerging themes. After the initial coding, the framework underwent a pilot test on 2 interviews, independently conducted by all researchers (AM, RB, and SA). Any discrepancies or differences were resolved, and the finalized framework was applied to all transcripts. In the second stage, descriptive themes were developed by grouping codes with similarities. For example, codes were grouped if caregivers continued with challenges or positive changes post COVID-19. Coding the data regarding the ways in which COVID-19 continues to impact their lives was instrumental to understanding the caregivers’ experiences. This ensured consistency and relevance in the analysis. It should be noted that determining the broad themes prior to analysis did not influence the coding process of the interview transcripts, and all identified themes were able to be grouped within the socioecological model framework. In the third stage, relationships between the codes were synthesized into analytical themes within the levels of the socioecological framework.

An iterative process was employed during data interpretation, involving reflective practices and meetings between both researchers to discuss the coding and interpretation processes. Rigor and trustworthiness were ensured through measures such as member checking, reflective journaling, and reflective conversations between the 3 authors. The coding and analysis were based on the socioecological framework. The model was adopted to analyze the various levels of factors that caregivers of children with cerebral palsy experienced post pandemic.

## Results

### Profile of Participants

The number of caregivers contacted is unknown as this was through the different schools/organizations. However, 14 caregivers participated in the study (2 were from the same family). Twelve children with cerebral palsy were represented in this sample. Majority of participants were female (n* *= 13). Participants interviewed were predominantly mothers (n* *= 9), with some also identifying as grandmothers (n* *= 2), a sister (n* *= 1), a father (n* *= 1), and an aunt (n* *= 1) (see Table 1 for participant demographics). It is important to highlight the gendered composition of the sample and the caregiving role that is often predominantly female, particularly in the South African setting. As a result, many women often experience a multidimensional caregiving burden regarding additional stressors around poverty and well-being.^22,23^ Participants had varying types of employment, most of whom were employed (n* *= 9), with 5 participants being unemployed at the time of the study and 1 participant continuing to experience unemployment due to job loss as a result of the COVID-19 pandemic (participant 11). Of the participants, 4 had access to private health care, and 5 had access to public health care only. Five participants reported having access to both public and private health care. However, it is important to note that these participants predominantly used public health care services and accessed rehabilitation services privately. The participant information highlights the heterogeneity of the sample and the diversity within the South African context.

**Table 1.** Demographic Data of Caregivers (n* *= 14).

Children with CP	Mean (SD) or n	Range or %
Age, y	38.5 (9.1)	22-59
Gender, n (%)		
Male	1	7
Female	13	93
Relationship to child, n (%)		
Mother	9	64
Father	1	7
Aunt	1	7
Grandmother	2	14
Sister	1	7
Employment, n (%)		
Employed	9	64
Unemployed	4	36
Relationship status, n (%)		
Single	6	43
Married	6	43
Divorced	1	7
In a relationship	1	7
Public or private health care, n (%)		
Public	5	36
Private	4	26
Both	5	36

Abbreviation: CP, cerebral palsy.

All families had a child with cerebral palsy between the ages of 4 and 12 years (mean = 7.3 years, standard deviation [SD] = 2.6), with slightly more males (n = 7) than females (n = 5). Caregivers reported their child's ambulatory status according to the Gross Motor Function Classification System level. It is important to note that these were caregiver interviews and that the children were not formally assessed. Among the children, 36% were ambulatory (levels I, II, and III), and 50% were nonambulatory (levels IV and V) (Table 2).

**Table 2.** Demographic Data of Children With Cerebral Palsy (n* *= 12).

Children with CP	Mean (SD) or n	Range or %
Age	7.3 (2.6)	4-12
Gender, n (%)		
Male	7	58.3
Female	5	41.7
GMFCS class, n (%)		
Level 1	0	0
Level 2	0	0
Level 3	5	41.7
Level 4	6	50
Level 5	1	8.3

Abbreviations: CP, cerebral palsy; GMFCS, Gross Motor Function Classification System.

**Figure 1.**
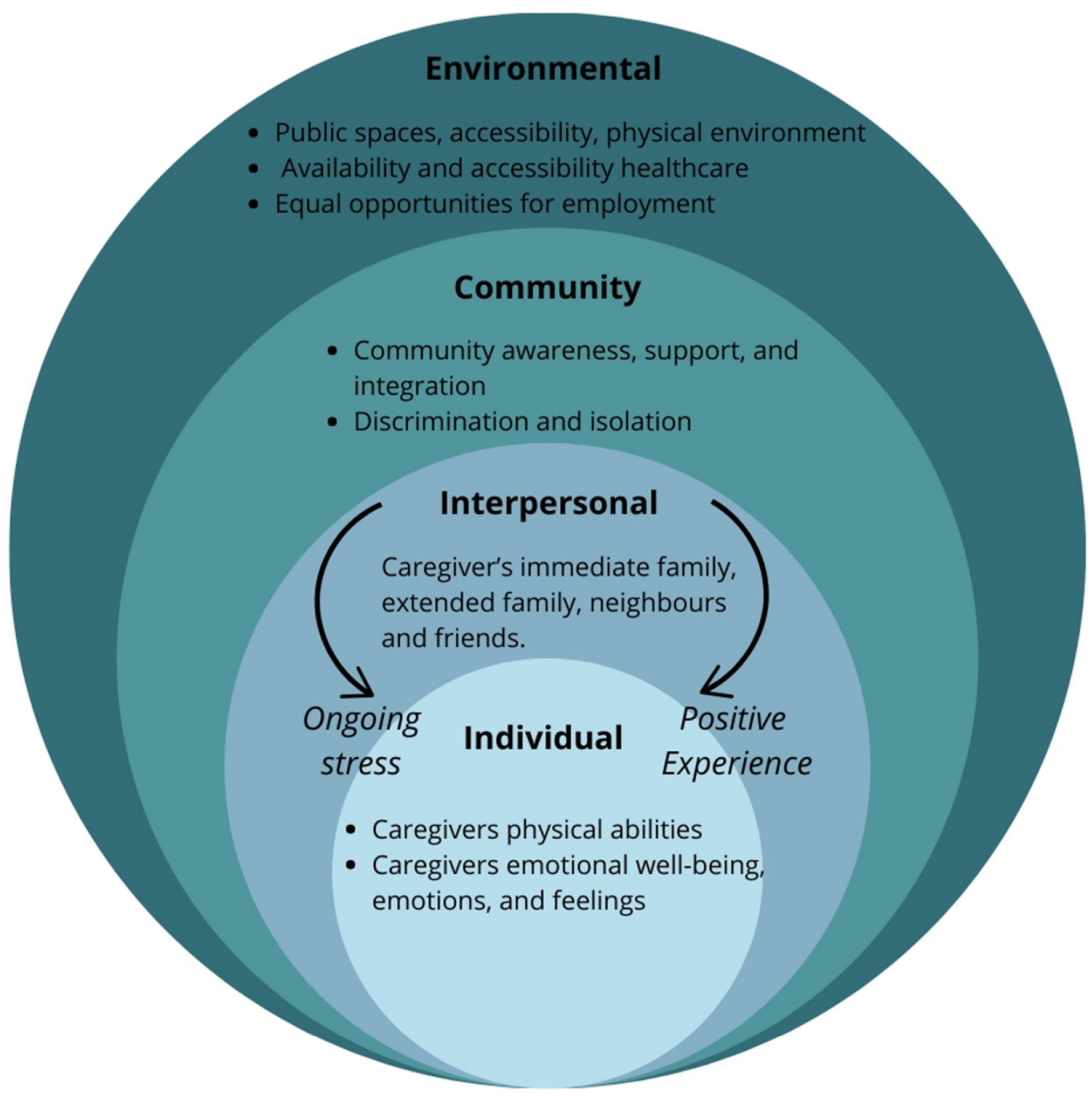
Adapted socioecological model of factors of the different post-pandemic factors classified.

The results are presented under the various dimensions of the socioecological framework as shown in Figure 1.^24^ Following the analysis, several core themes emerged from the data illustrating the added challenges experienced by caregivers of children with cerebral palsy, some of the positive changes that have happened as a result of the pandemic, and the need for additional and continued support. Coding and analysis were based on the socioecological framework in order to analyze the various levels of factors that may be represented as continued challenges, positive outcomes, and continued supports. Verbatim participant quotes are included to support the themes identified.

## Individual Factors

These are individual-level factors from the family members’ responses to support the themes identified related to their physical abilities, emotional well-being, emotions, and feelings.

### Physical Stressors

The physical strain included activities such as feeding, bathing, and carrying the child, often exacerbated by the child's increased weight because of the lockdown's sedentary lifestyle. As children were at home, caregivers were required to take on all the care for their child, which had a significant impact on their physical well-being. Participants spoke about how their children gained a significant amount of weight that has continued to physically impact on the caregiver, “My child also grew a lot in lockdown and would not fit in his buggy so I had to carry (child) on my back and he is heavy so it was a bit of a struggle” (participant 4). Caregivers struggle with the added weight gain due to their need to constantly lift, bend, carry, and reposition their child. This has placed significant stress on the caregiver's back, neck, and legs as mentioned by participant 11, “I would have to carry (child) to move him around. My body was always aching, and my back was always sore, and my knees give in often. It still hurts.” As a result, many caregivers have continued to experience body aches and pains.

### Emotional Stressors

Four participants mentioned the continued impact that the lockdown had on their emotional health and well-being spending more time with their child during the lockdown, “Emotionally it was a lot I don't want to lie, being in the same space it hit me that my grandchild has a disability. I needed therapy. I feel like she needed therapy, you know, and this was someone I was used to just seeing in the evenings and weekends but now because it was locked down she was with me every day. After the lockdown I am still worried about her disability and it can depress me but now with some time it is getting better” (participant 10). Participants spoke about the continued emotional impact of COVID-19 and how psychological support is still required, “During the lockdown I felt so guilty and I still do today because he already gained weight cause of no moving, no exercise, nothing. Then he's heavy changing Pampers you know, it affects you. You worry what you did” (participant 2). Participant 11 had a similar experience where she felt guilty that her son only watched television during lockdown, “The only difficult thing was trying to keep him busy when he's not watching cartoons because if he does not have anything to do he gets frustrated. So he would just watch cartoons. Now at least (child) is back at school, but it makes me feel like a bad mother.” Many mothers also experienced emotional stress as a result of the blame that they placed on themselves for not being able to do as much as they thought they should be doing.

### “After Lockdown, We Are Now Free”

All 14 caregivers interviewed reported improved experiences after the lockdown because of their child now being able to attend school again and they now have a sense of freedom, “After lockdown, we are better than during lockdown. During lockdown, we like, there was no life. We were just in prison. Then after lockdown, we are now free” (participant 1). Participant 5 further reported on her excitement that the lockdown has ended and that her child is no longer dependent on only her, “I'm alive. No, I feel like I'm alive again. During lockdown my child struggled in every way, like I can just say most of the things because he's not talking, he's not sitting, he's not standing, he's not walking. So he's total dependent on me. So everything, feed him, bathe him, clean him. So everything a hundred percent depends on me.” Participant 4 described how during the lockdown she struggled with spending so much time with her child and not knowing how to respond or why he was upset but feels she can manage better now that the lockdown has ended, “It was challenging. It wasn't positive. It was challenging and frustrating. He'll be crying and sometimes when he cries, it frustrate [*sic*]. Sometimes you now be forced, like, let me hit him. You don't know what to do. You get confused. Now, lockdown is over, I am out the house and I have more patience for my child and trying to understand what he wants.”

## Interpersonal

Participants’ interpersonal relationships encompassed immediate family members, extended family, neighbors, and friends. These relationships were perceived by participants as either an ongoing source of stress or a positive influence arising from the pandemic.

### Absent Fathers

In the study there were many supportive fathers and husbands, although 6 participants did speak to the fact that the fathers were either uninvolved or absent before COVID-19. Additionally, 2 participants mentioned that even if fathers were initially engaged, this involvement ceased during the lockdown, with the fathers disengaging from their children and families as participant 4 remarked, “(Child's) father never really helped in lockdown because he now stays far and with curfew. So it was just me—and it still is. Only now again his father can take him for some time when I need to do something.” Participant 14 noted that her and the father got divorced after COVID-19 because of to the stressors of the pandemic and the resulting lack of support, “We got divorced in that period because it just, there wasn't anything we could do to salvage this marriage. I was relying on him and he was not helping out when I needed him to. And when he eventually moved out, he didn't even tell me, “Okay, I'm moving out today.” I just came home after being at my sister's house on the weekend; when I got home, he was gone.”

### Continued and Exacerbated Social Exclusion

Eight caregivers reported continued isolation from society because of the stigma around children with disabilities. Because of COVID-19, people are more concerned about the possibility of contracting illnesses from others. As a result, they have become more cautious about their interactions with people in their surroundings. This led to isolation as others were hesitant to engage with the caregiver and the child, “Now I always feel excluded because now no one visits me or invites me anymore, even people that did before COVID. I think because I always said no during COVID” (participant 11).

### Bonding and Time Spent as a Family

Although the majority of the caregivers viewed the increased time spent with their child as a contributing factor to their emotional stress, caregivers also viewed the time spent with their child and their family as a positive, and that one can always appreciate the time spent with family during the lockdown because it made them closer postlockdown, “So we definitely did get to spend time together and we still do now. So the time aspect is, we can always appreciate the time aspect” (participant 6). “I think we became close as a family and we learned to appreciate each other and we had time for each other, I can understand my child's needs better. Even if he can’t talk, now I know what he wants” (participant 4).

## Community

Community factors extend beyond individual relationships to the broader social environment. This dimension encompasses the physical, emotional, social, and financial aspects within the community that impact caregivers and children with cerebral palsy. It includes community services, awareness, and social integration opportunities.

### Discrimination

Caregivers reported stigma or fear associated with the perceived vulnerability of children with cerebral palsy to illnesses, including post-pandemic concerns. As mentioned by participant 6, “People are ignorant, they don’t understand what cerebral palsy is, and they aren’t open to accepting that. During lockdown my child was at home a lot and frustrated. Some [neighbors] will come, they'll be complaining, your child is making noise, your child is crying, is screaming, and it is not easy. He's not accepted, and they still complain.” Participant 4 expressed similar concerns, “They won't even touch your child's hand. They won't even say hello. My grandchild's disability is not contagious.”

## Environmental

The environmental dimension focuses on the physical and emotional aspects of the surrounding environment. This includes accessibility to public spaces, the emotional impact of the physical environment, and the influence of social norms within the community.

### Continued Challenges With Access

Access to medical treatment and care continues to be a problem post COVID-19, which is a specific concern for children with cerebral palsy who may have experienced increased stiffness and regressions during the pandemic, “Access to medical is a big problem even after Covid. He's stiff, all the muscles are stiff, nothing, he can't move them. And I'm the one going through, he's in pain, crying. Pains are all over and there is nothing I can do” (participant 2). Participant 14 spoke about the halt in therapy sessions during lockdown and the continued challenges in resuming them and starting with a new therapist, “During the hard lockdown, we didn't have therapy sessions. When we started again her old therapist had left and we got no handover. She started with a new therapist. So we were just basically handed over to another therapist.”

### Employment Difficulties and Continued Financial Strain

A central concern reported by the majority of the caregivers includes the financial burden that worsened during the lockdown and still continues. Participant 11 expressed the harsh impact that the lockdown had on her employment, “The most difficult thing of lockdown that was a change for me was that I lost my job and have been unemployed since then.” Participant 11 further expressed the continued impact that the unemployment has had on her financial status. Participant 1 expressed similar concerns stating, “What I feel that we are still struggling with because of lockdown is financially, cause things have not gone, they are not the same. Caring for my child costs as much as a new car.” This highlights the increased financial support needed by caregivers because of job loss and continued unemployment caused by COVID-19.

## Discussion

This study identified important factors within the socioecological framework that have affected caregivers of children with cerebral palsy and their transition back to normalcy following the end of the pandemic in the South African context. The study by Vadivelan et al^3^ explored the burden of caregivers of children with cerebral palsy within the domains of the individual, family, community, and environmental that were explored.^17^ Although some of the findings are similar to the experiences of caregivers of children without disabilities during the pandemic,^6,7,11,14^ this study highlights the unique and ongoing challenges faced by caregivers of children with cerebral palsy. Although both groups experienced significant disruptions and the need for continued support, this research delves deeper into the long-term effects of COVID-19 on caregivers of children with cerebral palsy. Notably, the study highlights the significant challenges that emerged because of COVID-19 restrictions, including heightened physical pain, ongoing emotional distress, and a lack of social and governmental support. This lack of support encompassed financial assistance and sustained unemployment and unrecompensed expenses incurred during COVID-19. These long-term impacts have continued to affect families, underscoring the need for targeted, ongoing support.

The theoretical assumption posits that caregiver burden is affected by the intersectionality of the characteristics of the caregivers, interpersonal relationships, social interactions, social support, organizational structure, and the environment, as outlined by the socioecological model of health used in this study. According to this model, the health of an individual is shaped by multiple layers, encompassing influences at the individual level, interpersonal relationships, social factors, and environmental influences.^24^ Caregivers of children with cerebral palsy experienced a number of physical and emotional stressors that they have continued to experience since the end of the pandemic.^25^ Caregivers faced significant challenges as their children were unable to participate in physical activities. This, combined with the child's advancing age, weight gain, and the increased responsibility of taking over all caregiving tasks, placed heightened strain on caregivers during and after the lockdown period. Previous research has highlighted that children with cerebral palsy are often less active than their typically developing peers, and during COVID-19, their physical activity levels significantly declined because of lockdown restrictions.^26,27^ Although many children gained weight and grew larger during this time, this was not solely because of COVID-19 but was exacerbated by the sudden stop in physical activities.^26^ In addition, many caregivers expressed guilt and frustration at having to care for their child and feeling as though they were not able to do enough during lockdown. Building on the existing literature, this study offers findings that strongly align with research that was conducted during COVID-19 but has highlighted the continued challenges experienced.^11,14,28,29^ The cumulative effects of these challenges may have long-term implications for the overall well-being of both the caregivers and the children they support.

The end of lockdown restrictions allowed caregivers to regain a sense of freedom and the caregivers felt less confined. This shift from confinement to freedom proved to be meaningful for caregivers who had been isolated for an extended period because of the lockdown. October et al^30^ found that caregivers in South Africa reported an improved overall experience after the COVID-19 lockdown; this aligns with the findings of this study, which further explored the experiences of caregivers of children with cerebral palsy. The results of the study indicate that both during and after the lockdown, the daily routines and family events of households were significantly altered both within their internal dynamics and in relation to their external family environment. Many caregivers reported on the continued social exclusion, with 2 caregivers also describing how uninvolved or absent the fathers were both during and after COVID. These were defined as interpersonal problems that exacerbated participant stress and are similar to those reported in previous literature.^2,31^ However, it is important to note that following COVID-19, some families also reported on additional bonding with their child and the support provided by the fathers and other family members. Positive interpersonal relationships and changes have been reported in previous literature as an outcome of the COVID-19 lockdown.^11^ Therefore, it is critical that the ways in which caregivers have positively experienced the end of lockdown are cultivated and supported to ensure improved emotional well-being using interpersonal support systems and development of new relationships, particularly because caregivers who have immediate family support usually have better emotional well-being.^2,31^

This study did not only explore the experiences of the primary caregiver and unique insights were offered into the experiences of additional caregivers such as aunts, grandmothers, sisters, and fathers. The findings revealed a commonality in experiences among all caregivers, specifically in terms of the physical and emotional challenges associated with caregiving. This study makes findings unique to the context regarding the primary sample of women who are the primary caregivers. This observation emphasizes the need to extend the exploration of caregiving experiences to include other family members within the South African context. It is noteworthy that many children in South Africa are not solely raised by their mothers, and acknowledging and addressing the unique challenges faced by different caregivers is crucial.^32^ It is important to acknowledge that in general, women may be more vulnerable in these societies, with some coming from lower socioeconomic backgrounds.^23^ In addition, the intersection of gender and poverty puts these women at high levels of vulnerability.^19,20^ Furthermore, the high level of vulnerability is further compounded by the burden of caring for a child with a disability, and the experience of COVID-19. Therefore, support and interventions should be tailored to accommodate the diverse caregiving roles within the family structure, particularly women caregivers.

There are several limitations of our study to be considered. First, a small geographical location was targeted in this research, which was seen as a limitation because experiences were limited to caregivers in Gauteng. Additionally, the study primarily addressed an urban population, overlooking the perspectives of caregivers in low-resourced contexts, which is a limitation as this demographic is often underrepresented in existing literature. To ensure comprehensive support for all those in need and still grappling with the aftermath of the pandemic, further investigation should include engagement with children and caregivers facing other neurodevelopmental disabilities. Further research is also needed on evidence-based psychological interventions to minimize the negative impact of the pandemic on caregivers’ mental health.

## Conclusion

Our findings add to the growing body of literature on the impact COVID-19 has had on children with disabilities as well as associated risks, stressors, and provisions that need to be made for this population. Caregivers of children with cerebral palsy have experienced significant challenges because of COVID-19, as well as continued difficulties even after the end of the pandemic. This research emphasizes the continued impact of caregiver challenges even after the pandemic has ended. By focusing on these persistent difficulties, the study adds a nuanced understanding of the long-lasting effects on caregivers and the need for ongoing support beyond the initial crisis. This knowledge can guide the development of public health policies aimed at supporting caregivers and enhancing their overall quality of life.

## Supplemental Material

sj-docx-1-jcn-10.1177_08830738241292844 - Supplemental material for A Socioecological Framing of the Experiences of Caregivers of Children With Cerebral Palsy in South Africa Post COVID-19 Supplemental material, sj-docx-1-jcn-10.1177_08830738241292844 for A Socioecological Framing of the Experiences of Caregivers of Children With Cerebral Palsy in South Africa Post COVID-19 by Skye Adams, Aneesah Moosa and Razina Bhorat in Journal of Child Neurology

sj-docx-2-jcn-10.1177_08830738241292844 - Supplemental material for A Socioecological Framing of the Experiences of Caregivers of Children With Cerebral Palsy in South Africa Post COVID-19 Supplemental material, sj-docx-2-jcn-10.1177_08830738241292844 for A Socioecological Framing of the Experiences of Caregivers of Children With Cerebral Palsy in South Africa Post COVID-19 by Skye Adams, Aneesah Moosa and Razina Bhorat in Journal of Child Neurology
