# Supplementary material for: A Socioecological Framing of the Experiences of Caregivers of Children With Cerebral Palsy in South Africa Post COVID-19
Source: J Child Neurol. 2024 Nov 26;40(3):191–9. doi: 10.1177/08830738241292844 (PMC11909768; doi:10.1177/08830738241292844)
Supplement: sj-docx-1-jcn-10.1177_08830738241292844 - Supplemental material for A Socioecological Framing of the Experiences of Caregivers of Children With Cerebral Palsy in South Africa Post COVID-19 [file sj-docx-1-jcn-10.1177_08830738241292844.docx]

**Supplementary material 1**

**Demographic questionnaire:**

**Participant number:**

**Participant details:**

1. Age:
2. Relationship to child:
3. Where do you live (geographical location e.g. Johannesburg, Pretoria):
4. Members living at home:
5. Are you the main member who takes care of your child? If no, who assists you?
6. Do you have other children? If yes, how many?
7. Present occupation:
8. If unemployed, are you seeking employment?
9. Were you employed before the COVID-19 lockdown? If yes, what was your occupation?
10. Were you working from home during the COVID-19 lockdown? If yes, who would look after your child during that time?
11. Did your child attend online school or therapy during the lockdown?
12. How you do you move around with your child? (pram, carrier, car, bus, taxi etc)
13. Private/public healthcare access:
14. **Child details:**
15. Type of CP:
16. GMFCS classification (if known):
17. Age of child:
18. Gender of child:

**Interview Guideline**

Try to achieve in-depth answers. Example questions to ask open-ended supplementary questions:

- Can you explain that?
- What do you mean by that?
- Can you give an example?

**Individual:**

1. Tell me about your experiences of the lockdown
   1. How did the lockdown impact you and your child?
   2. Were there any additional challenges noted during the lockdown period?
   3. What were your daily activities during lockdown?
2. Are there any aspects of your daily life that has changed since the lockdown has ended?
   1. What continued challenges have you experienced?
   2. Can you describe any positive experiences since the end of the lockdown?
3. How would you compare life during lockdown and now that restrictions have been lifted?
4. Are you satisfied with your daily life at this moment
   1. Are there any aspects you would like to change?

**Interpersonal:**

1. What type of support do you still receive now that the lockdown is over?
   1. How did these people support you?
   2. Can you describe any difficulties with access to support?
   3. What support do you think you still require
   4. How has your support systems changed now that the lockdown is over?
2. How has the end of the lockdown impacted your family life?
   1. What do you feel you still struggle with as a result of the lockdown?
   2. What positive changes have occurred as a result of the lockdown?

**Community:**

1. Do you feel supported in your community, and how so?
2. Is it easy to get around your neighbourhood of your home?
3. Are there any barriers or facilitators that you encounter in the neighbourhood of your home since the end of the lockdown that were not there before.

**Environmental:**

1. What are the programs or policies or things that government has done that has shown continued support since the end of the lockdown.
2. What are your recommendations regarding services and support for you and your child would you like to see more of?
